# Digital health literacy for COVID-19 vaccination and intention to be immunized: A cross sectional multi-country study among the general adult population

**DOI:** 10.3389/fpubh.2022.998234

**Published:** 2022-09-16

**Authors:** Roy Rillera Marzo, Tin Tin Su, Roshidi Ismail, Mila Nu Nu Htay, Mohammad Yasir Essar, Shekhar Chauhan, Mark E. Patalinghug, Burcu Kucuk Bicer, Titik Respati, Susan Fitriyana, Wegdan Baniissa, Masoud Lotfizadeh, Farzana Rahman, Zahir Rayhan Salim, Edlaine Faria de Moura Villela, Kittisak Jermsittiparsert, Yadanar Aung, Nouran Ameen Elsayed Hamza, Petra Heidler, Michael G. Head, Ken Brackstone, Yulan Lin

**Affiliations:** ^1^Department of Community Medicine, International Medical School, Management and Science University, Kuala Lumpur, Malaysia; ^2^Global Public Health, Jeffrey Cheah School of Medicine and Health Sciences, Monash University Malaysia, Bandar Sunway, Malaysia; ^3^Department of Public Health, Faculty of Medicine, Asia Metropolitan University, Johor, Malaysia; ^4^South East Asia Community Observatory, Monash University Malaysia, Bandar Sunway, Malaysia; ^5^Department of Community Medicine, Faculty of Medicine, Manipal University College Malaysia, Melaka, Malaysia; ^6^Department of Dentistry, Kabul University of Medical Sciences, Kabul, Afghanistan; ^7^Department of Family and Generations, International Institute for Population Sciences, Mumbai, India; ^8^School of Criminal Justice Education, J. H. Cerilles State College, Caridad, Philippines; ^9^Department of Medical Education and Informatics, Faculty of Medicine, Gazi University, Ankara, Turkey; ^10^Faculty of Medicine, Universitas Islam Bandung, Bandung, Indonesia; ^11^College of Health Sciences/Nursing Department, Sharjah Institute of Medical and Health Sciences, University of Sharjah, Sharjah, United Arab Emirates; ^12^Department of Community Health, Shahrekord University of Medical Sciences, Shahrekord, Iran; ^13^Administration and Research, Bangladesh National Nutrition Council, Ministry of Health and Family Welfare, Dhaka, Bangladesh; ^14^College of Business Administration, International University of Business Agriculture and Technology, Dhaka, Bangladesh; ^15^Public Policies, Education and Communication, Disease Control Coordination, São Paulo State Health Department, São Paulo, Brazil; ^16^Faculty of Education, University of City Island, Famagusta, Cyprus; ^17^Publication Research Institute and Community Service, Universitas Muhammadiyah Sidenreng Rappang, South Sulawesi, Indonesia; ^18^Sekolah Tinggi Ilmu Administrasi Abdul Haris, Makassar, Indonesia; ^19^Medical Statistics Division, Department of Medical Research, Ministry of Health, Nay Pyi Taw, Myanmar; ^20^Medical Agency for Research and Statistics, Giza, Egypt; ^21^Clinical Research Key (CRK-CRO), Nairobi, Kenya; ^22^Department for Economy and Health, University for Continuing Education Krems, Danube University Krems, Krems an der Donau, Austria; ^23^Department of Health Sciences, St. Pölten University of Applied Sciences, St. Pölten, Austria; ^24^Clinical Informatics Research Unit, Faculty of Medicine, University of Southampton, Southampton, United Kingdom; ^25^Department of Epidemiology and Health Statistics, School of Public Health, Fujian Medical University, Fuzhou, China

**Keywords:** COVID-19, health literacy, vaccine intention, multi-country, digital

## Abstract

**Introduction:**

It is clear that medical science has advanced much in the past few decades with the development of vaccines and this is even true for the novel coronavirus outbreak. By late 2020, COVID-19 vaccines were starting to be approved by national and global regulators, and across 2021, there was a global rollout of several vaccines. Despite rolling out vaccination programs successfully, there has been a cause of concern regarding uptake of vaccine due to vaccine hesitancy. In tackling the vaccine hesitancy and improving the overall vaccination rates, digital health literacy (DHL) could play a major role. Therefore, the aim of this study is to assess the digital health literacy and its relevance to the COVID-19 vaccination.

**Methods:**

An internet-based cross-sectional survey was conducted from April to August 2021 using convenience sampling among people from different countries. Participants were asked about their level of intention to the COVID-19 vaccine. Participants completed the Digital Health Literacy Instrument (DHLI), which was adapted in the context of the COVID Health Literacy Network. Cross-tabulation and logistic regression were used for analysis purpose.

**Results:**

Overall, the mean DHL score was 35.1 (SD = 6.9, Range = 12–48). The mean DHL score for those who answered “Yes” for “support for national vaccination schedule” was 36.1 (SD 6.7) compared to 32.5 (SD 6.8) for those who either answered “No” or “Don't know”. Factors including country, place of residence, education, employment, and income were associated with the intention for vaccination. Odds of vaccine intention were higher in urban respondents (OR-1.46; C.I.-1.30–1.64) than in rural respondents. Further, higher competency in assessing the relevance of online information resulted in significantly higher intention for vaccine uptake.

**Conclusion:**

Priority should be given to improving DHL and vaccination awareness programs targeting rural areas, lower education level, lower income, and unemployed groups.

## Introduction

The COVID-19 pandemic, in which confirmed cases first appeared in China and the outbreak quickly has spread across the globe, was defined as a Public Health Emergency of International Concern on January 30, 2020. The World Health Organization (WHO) officially declared a pandemic on March 11, 2020. The pandemic has since resulted in a significant level of excess deaths and a huge socio-economic impact on countries around the world. During the earlier phases of the pandemic, many countries implemented precautionary measures such as mask wearing, quarantines, and curfews to slow the spread of the virus. These measures were effective in reducing both transmission and the overall burden of COVID-19 disease (1). However, research has conclusively shown that COVID-19 has severe health effects including quality of life (2), mental health (3–7), and psychological distress (8–13).

Since the start of the first outbreak in early 2020, there has been significant commentary about COVID-19 on social media, in which users have been exposed to good and bad quality information about the virus and the emerging outbreaks. In light of the significant amount of false information coming from digital platforms amidst the pandemic, the WHO introduced a new term—an infodemic—defined as “too much information including false or misleading information in digital and physical environments during a disease outbreak”. The WHO urged all nations to combat the COVID-19 infodemic (1).

By late 2020, COVID-19 vaccines were starting to be approved by national and global regulators, and across 2021, there was a global rollout of several vaccine candidates, including those manufactured by Pfizer and AstraZeneca. Since then, countries have urgently attempted to reach their populations and achieve high vaccine uptake. Mortality rates and cases numbers have fallen dramatically as a result of vaccination (14, 15). By reducing the pressures on national and local health services, immunization programs have helped the countries in easing down restrictions, and enabled people to resume their normal lives (16). Nevertheless, the virus is still highly prevalent around the world, with new variants fueling transmission, and too many people still awaiting access to even their first dose of any COVID-19 vaccine. As a result of misinformation and vaccine efficacy, vaccine hesitancy also plagued the global COVID-19 vaccination program (16–18). The acceptance of vaccine was further influenced by the recommendations of medical professionals (19, 20).

Vaccine hesitancy has been an important area of concern when considering pandemic response strategies. The main cause of vaccine hesitancy is misinformation that affects decision-making and causes hesitation in vaccination uptake (17). In a systematic review conducted by Cascini et al., a negative association between use of social media and people's intention to vaccinate themselves have been observed (21). Decisions to receive vaccination has been greatly impacted by the exposure to false information on social media. A further study conducted in Ghana, shows the influence of social media on the people's belief about vaccination (22). Additionally, people in Southeast Asia have become hesitant to vaccination due to the existence of misinformation through digital platforms (23). It is therefore important to acknowledge the use of social media as a tool through which misinformation can easily be spread. On the other hand, Morocco, a country located in North Africa has used its digital system to run a smart vaccination campaign. This digital system comprises a vaccination registry, stock, logistics management facilities, and a portal for tracking side effects of the vaccine. In addition, a new platform named “liqah” (“vaccine” in “Arabic”) has been established, which allows doctors to communicate directly with the citizens. The website also shares comprehensive information on the vaccines for the citizens (24). In a study conducted in eight European countries, it is shown that digital technologies and tools have supported the vaccination programs. Digital tools were used to convey information about the safety and efficacy of vaccines and how to access vaccine services (25). Digital health tools can also help with vaccine hesitancy. In order to do this, information from the platform should be conveyed in multiple languages, clearer language, and in a friendly manner. Moreover, the engagement platforms should be trustworthy and provide greater details for people who are in the greatest need of vaccine. Additionally, digital health tools should be inclusive and embrace all races and ethnicities (26).

People are increasingly using electronic resources to make decisions about their health, including social media, demonstrating the importance of digital health literacy (27). The WHO defines Digital Health Literacy as the ability to utilize electronic devices to gain, seek, appraise, and comprehend health information in order to improve health outcomes or address a health concern (28). Digital platforms are ideal places to communicate accurate information about COVID-19. However, social media platforms have become a hub of misinformation negatively impacting people's lives and attitudes concerning the pandemic. Monitoring digital platforms is essential toward ensuring that people have access to the best possible information at the appropriate time. To assess digital health literacy and its relevance to the COVID-19 vaccination, a cross-sectional study was conducted in 11 countries among the general adult population.

## Methods

### Study design

An internet-based cross-sectional survey was conducted from April to August 2021 using non-random convenience sampling among people from different countries.

### Data collection procedures

The sample size is estimated with an infinite population, a confidence level of 95%, a Z score of 1.960, and a margin error of 0.05. We distributed the Google form online without restriction for the specific country using personal contacts by emails, web-based applications (e.g., WhatsApp and Telegram), and social media (e.g., Facebook, LinkedIn, Twitter, and Instagram); over 4,700 subjects completed the surveys. Participants confirmed that they were aged 18 years or older. They were reminded to respond only once and use a unique identifier to create a single account by settings that allow one response per user. Finally, personal data protection was emphasized during the study to secure our data's privacy, availability, and integrity. Confidentiality and privacy of participants' responses were ensured to minimize potential bias caused by self-reported data. Data were collected using the online Google Forms platform. The collected information was exported for review in Microsoft Excel before a fuller analysis using Stata 16 (College Station, TX: StataCorp LLC).

### Instrument development and measures

The questionnaire was adapted from the World Health Organization's (WHO) survey tool and guidance on COVID-19 (29). This survey tool monitors knowledge, risk perceptions, preventive behaviors including digital health literacy, and other variables to inform COVID-19 outbreak response measures, including policies, interventions, and communications.

#### Demographics

Data collected included socio-demographic characteristics of participants, including as age, gender, education (secondary or less/post-secondary/tertiary), country of residence (the focus being Bangladesh, Brazil, Egypt, Indonesia, Iran, Malaysia, Myanmar, Philippines, Thailand, Turkey, United Arab Emirates, others), religion (Islam, Buddhism, Christianity, Hinduism, others), community type (rural/urban), employment status (working/not employed/unemployed/student), and income (self-reported as sufficient/less sufficient).

#### Vaccine intention

Participants were asked about their level of intention to the COVID-19 vaccine (“I think everyone should be vaccinated according to the National vaccination schedule”; no, I don't know, yes).

#### Digital health literacy

Participants completed the Digital Health Literacy Instrument (DHLI) (8), which was adapted in the context of the COVID Health Literacy Network. The scale measures one's ability to seek, find, understand, and appraise health information from digital resources. While the original DHLI is comprised of 7 subscales, this study used the following four domains: (1) information searching or using appropriate strategies to look for information (e.g., “When you search the internet for information on coronavirus virus or related topics, how easy or difficult is it for you to find the exact information you are looking for?”) (2) adding self-generated content to online-based platforms (e.g., “When typing a message on a forum or social media such as Facebook or Twitter about the coronavirus a related topic, how easy or difficult is it for you to express your opinion, thought, or feelings in writing?”) (3) evaluating reliability of online information (e.g., “When you search the internet for information on the coronavirus or related topics, how easy or difficult is it for you to decide whether the information is reliable or not?”) and (4) determining relevance of online information [e.g., “When you search the internet for information on the coronavirus or related topics, how easy or difficult is it for you to use the information you found to make decisions about your health (protective measures, hygiene regulations, transmission routes, risks and their prevention)?”]. A total of 12 items (three per each dimension) were asked, and answers were recorded on a four-point Likert scale (1 = *very difficult*; 4 = *very easy*). The reliability statistics (Cronbach alpha) for the overall DHL score was 0.92 while the alpha coefficients for the four subscales ranges from 0.73 to 0.88, suggesting acceptable to good internal consistency (30). Only participants who had complete data on all DHL subscales were included in the final analysis.

### Ethics statement

The study was designed and conducted in line with the declaration of Helsinki and was approved by the Asia Metropolitan University Ethics Committee in Malaysia (Ref. No: AMU/MREC/NF/18022021). Respondents were informed that their participation was voluntary, and written consent was implied on the completion of the questionnaire. All participants were aged 18 years or older.

### Statistical analysis

Descriptive analysis was conducted for socio-demographic variables. Continuous variables were presented as mean (standard deviation, SD). The outcome variable, vaccine intention, were dichotomized to “Yes” and “No/Don't know” while the DHL sub-scales and overall scores were dichotomized to “sufficient” vs. “limited” by median split in the analysis. Bivariate analyses between the socio-demographic variables and the DHL variables, and the vaccine intention were displayed using cross-tabulations and Chi-squared statistics were reported for statistical significance (*p* < 0.05). Multivariable logistic regression with robust variance were used to see associations between DHL overall (model 1) and DHL subscales (model 2) with vaccine intention, adjusted for age, sex, education, country, urban/rural, employment status and income. The variable “religion” was not included in the final models due to multicollinearity. Assumptions for logistic regression were met and multicollinearity was checked using variation inflation factor (VIF). Adjusted odds ratios (AOR, 95%CI) were reported in the models with the Hosmer-Lemeshow test reported for model fit. All analyses were conducted using Stata 16 (College Station, TX: StataCorp LLC).

## Results

The survey was completed by 4700 participants from 53 countries. The mean age was 29.4 (SD = 11.9 years), with range of 18–77 years. The majority of respondents were 18–29 years old (66%), female (58%), had tertiary level education (70%), and from Malaysia (33%). Other socio-demographic characteristics of the participants are summarized in Table 1.

**Table 1:** Socio-demographic characteristics of respondents.

	***n***	**%**
**Age group**
18–29	3,115	66.3
30–49	1,141	24.3
50 and above	444	9.4
**Gender**
Male	1,983	42.2
Female	2,717	57.8
**Education level**
Up to secondary	1,427	30.4
Tertiary	3,273	69.6
**Country**
Bangladesh	175	3.7
Brazil	140	3.0
Egypt	106	2.3
Indonesia	321	6.8
Iran	256	5.4
Malaysia	1,556	33.1
Myanmar	69	1.5
Philippines	919	19.6
Thailand	117	2.5
Turkey	586	12.5
United Arab Emirates	310	6.6
Other	145	3.1
**Religion**
Islam	2,723	57.9
Buddhism	412	8.8
Christianity	1,158	24.6
Hinduism	273	5.8
Other	134	2.9
**Community type**
Rural	1,546	32.9
Urban	3,154	67.1
**Employment status**
Working	2,047	43.6
Not working	1,314	28.0
Student	906	19.3
Other	433	9.2
**How sufficient do you consider your income?** ^**#**^
Sufficient	3,181	68.3
Less sufficient	1,480	31.8

# Missing income information, n = 39.

Overall, the mean DHL score was 35.1 (SD = 6.9, Range = 12–48). The mean DHL score for those who answered “Yes” for “support for national vaccination schedule” was 36.1 (SD 6.7) compared to 32.5 (SD 6.8) for those who either answered “No” or “Don't know”, *t*_(4,587)_ = 16.0, *p* < 0.001. The median for all the subscales scores were 9.0, 9.0, 9.0, and 9.0 (range 3–12), respectively, while the median for the total DHL score was 35.0 (range 12–48). The percentages of having “intention to get an immunization” within categories of socio-demographic characteristics and DHL sufficiency cut-off are displayed in Table 2.

**Table 2:** Bivariate associations between socio-demographic characteristics and sufficient DHL, with intention for vaccination.

	**“I think everyone should be vaccinated** **according to the national vaccination** **schedule”**				
	**Yes**		**No/don't know**		
	***n***	**%**	***n***	**%**	**χ^2^, *p*-value**
**Age group**					
18–29	2,113	67.8	1,002	32.2	109.409, *p* < 0.001
30–49	890	78.0	251	22.0	
50 and above	394	88.7	50	11.3	
**Sex**					
Male	1,468	74.0	515	26.0	5.259, *p* = 0.022
Female	1,929	71.0	788	29.0	
**Education level**					
Up to secondary	943	66.1	484	33.9	39.234, *p* < 0.001
Tertiary	2,454	75.0	819	25.0	
**Country**					
Bangladesh	100	57.1	75	42.9	745.275, *p* < 0.001
Brazil	134	95.7	6	4.3	
Egypt	70	66.0	36	34.0	
Indonesia	289	90.0	32	10.0	
Iran	198	77.3	58	22.7	
Malaysia	1,199	77.1	357	22.9	
Myanmar	47	68.1	22	31.9	
Philippines	410	44.6	509	55.4	
Thailand	107	91.5	10	8.5	
Turkey	574	98.0	12	2.0	
United Arab Emirates	196	63.2	114	36.8	
Other	73	50.3	72	49.7	
**Religion**					
Islam	2,152	79.0	571	21.0	381.538, *p* < 0.001
Buddhism	333	80.8	79	19.2	
Christianity	581	50.2	577	49.8	
Hinduism	232	85.0	41	15.0	
Other	99	73.9	35	26.1	
**Area of residence**					
Rural	970	62.7	576	37.3	104.509, *p* < 0.001
Urban	2,427	76.9	727	23.1	
**Employment status**					
Working	1,597	78.0	450	22.0	220.061, *p* < 0.001
Not working	819	62.3	495	37.7	
Student	748	82.6	158	17.4	
Other	233	53.8	200	46.2	
**How sufficient do you consider your income?**					
Sufficient	2,375	74.7	806	25.3	29.359, *p* < 0.001
Less sufficient	992	67.0	488	33.0	
**Sufficient DHL (total score)**					
Limited	1,286	63.0	754	37.0	166.543, *p* < 0.001
Sufficient	2,043	80.1	506	19.9	
**Subscale 1: Information seeking**					
Limited	1,066	61.4	669	38.6	161.694, *p* < 0.001
Sufficient	2,325	78.7	631	21.3	
**Subscale 2: Adding self-generated content**					
Limited	1,396	69.2	622	30.8	20.969, *p* < 0.001
Sufficient	1,951	75.2	642	24.8	
**Subscale 3: Evaluating reliability**					
Limited	1,294	63	761	37	159.181, *p* < 0.001
Sufficient	2,091	79.6	536	20.4	
**Subscale 4: Determining relevance**					
Limited	946	59.9	634	40.1	184.176, *p* < 0.001
Sufficient	2,441	78.6	663	21.4	

Missing values: Income (n = 39), total DHL score (n = 111), subscales 1 (n = 9), subscales 2 (n = 89), subscales 3 (n = 18), and subscales 4 (n = 16).

### Multivariable models

The multivariable logistic regression with robust variance models are shown in Table 3. The predictors of interest are sufficient (Sufficient vs. Limited) DHL score (Model 1) and each of the four subscales median cut-off (Model 2). After adjustment for age, sex, education, country, urban/rural, employment status and income, the Adjusted Odds Ratio (AOR) for intention to vaccination was 1.64 (95% CI, 1.41–1.90) for sufficient DHL. In Model 2, only subscale 4 (determining relevance) was a statistically significant factor for predicting intention to vaccination, AOR 1.48 (95% CI, 1.21–1.80).

**Table 3:** Adjusted ORs (95% CI) for sufficient total DHL score and sufficient DHL subscales scores in relation to “intention for vaccination”^#^.

	**Sufficient DHL (Model 1)**	**Sufficient DHL subscales** **(Model 2)**
Overall DHL	1.64^***^ (1.41, 1.90)	
Subscale 1: Information seeking		1.12 (0.92, 1.36)
Subscale 2: Adding self-generated content		1.10 (0.92, 1.30)
Subscale 3: Evaluating reliability		1.16 (0.95, 1.42)
Subscale 4: Determining relevance		1.48^***^ (1.21, 1.80)
Observations	4,553	4,553
Pseudo *R*^2^	0.181	0.185
Hosmer-Lemeshow chi-squared	8.38 (df = 8), *p* = 0.397	2.68 (df = 8), *p* = 0.953

Adjusted for age, sex, education, country, urban/rural, employment status. and income.

^#^The outcome variable is “intention for vaccination”, Yes = 1, No/Don't know = 0 (reference).

*** p < 0.001.

## Discussion

This study provides insights into digital health literacy and its association with the intention of vaccination across 53 countries, but with a predominant focus on 11 countries. To the best of our knowledge, this is the first study to investigate the DHL and intention to vaccinate during the COVID-19 outbreak in a wider geographical area. Our findings indicated that respondents had a high overall score of digital health literacy (*M* = 2.93, *SD* = 0.58). Similarly, sufficient DHL levels were reported among the university student population in the US (31), Germany (32), Pakistan (33), Malaysia, China, and the Philippines (34). Although inclusion criteria covered the general population, the respondents were predominantly younger adults, and approximately 70% attained tertiary education, which may explain the similar level of DHL levels with previous studies. For instance, older adults were associated with lower digital health literacy level, limited utilization of technology and electronic devices, and lower confidence in using technology (35). During this digital era with the increasing speed of utilization, digital information sources have tremendous potential benefits to the population's health (36). Thus, attaining a sufficient level of DHL is a positive prospect for positive health behaviors, including combating and preventing COVID-19 infection.

From the perspective of public health, improving health literacy among the population is considered a social vaccine to prevent, protect, and reduce the burden of diseases (37). During the COVID-19 pandemic, DHL is a critical tool to reduce the impact of the infodemic, and to improve the dissemination of high-quality pandemic-related information around topics such as preventive behaviors and vaccination (31, 33). Among our study respondents, those who supported being vaccinated according to the National Vaccination Schedule were found to have significantly higher digital health literacy scores compared to those who opposed this concept. Our findings are correlated with a previous study in the US, where a higher DHL level is associated with the willingness to have COVID-19 vaccination (38).

Among those who have sufficient DHL, some demographic factors were found to be associated with the intention to be vaccinated. In terms of geographic location, respondents from Turkey and Brazil reported having the significant highest intention compared to respondents from Bangladesh. The WHO are cooperating and collaborating with countries to ascertain equal access to the COVID-19 vaccination as it is the key factor to combat the pandemic (38). However, perception of vaccination, intention, and willingness plays a crucial role in the vaccine uptake during the pandemic. In a comparison across various countries, willingness to take the vaccination in low- and middle-income countries in Africa, South Asia, and Latin America was found to be an average of 80.3% in the previous study (39). In the UK, a similar finding of high willingness (88.8%) to take COVID-19 vaccination was reported (40). Meanwhile, in the US, 67% of the study respondents reported their willingness to vaccinate (41). Vaccine acceptance was found to be varied in previous studies across the UK, US, South Asia, Africa, and Latin America (39–41). Among the Canadian population, only 9% of the respondents in the nationwide survey reported that they had no intention to take COVID-19 vaccination (42). Different levels of intention for vaccine uptake across the countries might be contributed by the incidence of COVID-19 infection, public awareness level, and sampling recruitment in studies (43–46). Furthermore, willingness to take vaccination could be varied by contextual influence including politics and policies, individual and group influence, and vaccine-related factors such as the design and the delivery program, recommendations from healthcare personnel, and ability to understand (i.e., language and health literacy) (47). High willingness to vaccinate was reported among the Canadian community (42), where government policy was committed to vaccination by providing the financial, policy, and legislative support, by developing specific strategies for some groups including indigenous, pregnant, and persons with disabilities, minor ethnic groups and immigrants (48, 49). Political ideologies might also be related to vaccine uptake, as some states in the US achieved 70% vaccination, while another state reported only 35% of vaccination (49). Therefore, the local authorities need to understand the community perception, changes in that perception over time around the willingness to vaccinate, and should have a strong political commitment. This is important not only for the COVID-19 vaccine, but also for all other nationally recommended vaccines. The WHO and UNICEF have highlighted a global rise of measles outbreaks in the first quarter of 2022, as population mixing begins to return to pre-pandemic levels but also after 2 years of interrupted healthcare (50). Thus, a proactive approach to health promotion around routine vaccinations is important.

Respondents who achieved tertiary education were more likely to take the vaccine compared to those who achieved up to secondary education. Education has been reported as one of the influencing factors on intention and willingness to vaccinate in previous studies (51–54), albeit with occasionally conflicting results. For example, a study in Ghana found that higher education was linked to increased hesitancy, rather than increased willingness to vaccinate, with political allegiance likely to be a confounding variable when considering education (55).

The community needs trustworthy information about the disease, including the benefits of physical and mental wellbeing. Since the digital media is the major source of information, competency in online information-searching and evaluating the validity and reliability of information are associated with the utilization of trustworthy information sources (34). People with higher education levels may search for scientifically established information with critical evaluation compared to lower education group regards to COVID-19 related information and vaccination (56, 57). Furthermore, urban residents, employed people, and those who have sufficient income are positively associated with the intention to vaccinate. Previous studies reported that demographic factors such as residency and income influence knowledge, perceptions, and acceptance of COVID-19 vaccination (17, 58).

During the COVID-19 pandemic, people have also searched for health information *via* the traditional media, including television channels, national newspapers, government webpages (34, 59). Traditional channels play an essential role in providing informed and evidence-based vaccine-related content. In the meantime, there is potential for social media to educate people and reduce vaccine hesitancy (59). In this study, the key finding was that determining relevance of COVID-19 information was a significant factor regarding the intention to vaccinate. Moreover, the mean score for determining the relevance domain was found to be the highest among the DHL domains. Thus, when respondents searched for online information about the COVID-19 vaccines and related topics, most respondents found it to be easy to apply the online information in daily life, and used the online search results to make health-related decisions. This was ultimately associated with positive intentions toward vaccination. While developing vaccine-related information for online health communication strategies, key messages should be credible and relevant. Furthermore, the competency of people to determine the relevance and applicability to improve their health has an impact on the vaccination intention. Improving health literacy in the population and providing credible, timely, relevant information could enhance vaccination uptake in the community.

### Strength and limitations

To the best of our knowledge, this is the first to investigate the association between DHL and intention to vaccinate across 53 countries, with a focus on 11 countries in this paper. This study provides the current insight on vaccination intention across international context and highlighted the importance of DHL. Moreover, competency on determining relevance of information subscale in DHL was found to be particularly important for the willingness to take the vaccination.

Despite the strengths, our study has limitations. Since the non-probability method was used for recruitment, selection bias limits the generalizability of the findings. The nature of the cross-sectional study was to observe for a period of time; therefore, changes in vaccination intention and availability of vaccines could not be assessed. Approximately two-thirds of our respondents had tertiary education levels and reported living in an urban community, so the generalizability of the findings to other demographics such as rural populations and those of lower education level is uncertain. Considering that the level of digital health literacy is closely related to the internet penetration rate, level of economic development, reaching vulnerable individuals such as older adults, oversampling, and undersampling in some countries, the research results cannot represent the overall general adult population. Therefore, additional large-scale studies and a more systematic, inclusive sampling method are warranted to improve the representativeness and generalizability of the findings.

## Conclusion

This study provides an insight into the importance of DHL on the vaccination intention. The respondents generally have sufficient DHL competency. Among them, demographic factors, such as country, residence area, education, employment, and income were associated with the intention for vaccination. Higher competency in assessing the relevance of online information resulted in significantly higher intention for vaccine uptake. In terms of future perspective, not only for COVID-19 but also for the other vaccines, health promotion should be proactive in sharing relevant, timely and applicable information with the community. Priority should be given to improving DHL and vaccination awareness programs targeting lower education level, lower income, and unemployed groups.

## Data availability statement

The original contributions presented in the study are included in the article/Supplementary material, further inquiries can be directed to the corresponding authors.

## Ethics statement

The studies involving human participants were reviewed and approved by Asia Metropolitan University Ethics Committee in Malaysia (Ref. No: AMU/MREC/NF/18022021). The patients/participants provided their written informed consent to participate in this study.

## Author contributions

All authors made a significant contribution to the work reported, whether that is in the conception, study design, execution, acquisition of data, analysis and interpretation, or in all these areas, took part in drafting, revising or critically reviewing the article, gave final approval of the version to be published, have agreed on the journal to which the article has been submitted, and agree to be accountable for all aspects of the work.

## Funding

This work was supported by the Special Projects of the Central Government Guiding Local Science and Technology Development, China (No. 2021L3018). The funder was not involved in study design, in the collection, analysis and interpretation of data, in the writing of the manuscript, nor in the decision to submit the manuscript for publication.

## Conflict of interest

The authors declare that the research was conducted in the absence of any commercial or financial relationships that could be construed as a potential conflict of interest.

## Publisher's note

All claims expressed in this article are solely those of the authors and do not necessarily represent those of their affiliated organizations, or those of the publisher, the editors and the reviewers. Any product that may be evaluated in this article, or claim that may be made by its manufacturer, is not guaranteed or endorsed by the publisher.
