# Supplementary material for: Digital health literacy for COVID-19 vaccination and intention to be immunized: A cross sectional multi-country study among the general adult population
Source: Front Public Health. 2022 Sep 16;10:998234. doi: 10.3389/fpubh.2022.998234 (PMC9523876; doi:10.3389/fpubh.2022.998234)
Supplement: Supplementary file 1 [file Data_Sheet_1.docx]

**Appendix 1**

The scores for overall DHL items and the four DHL subscales were summarised in the table below. The mean (SD) for overall DHL score, “*information seeking*”, “*adding* *self-generated content”*, “*evaluating reliability” and “Determining relevance”* are shown below.

Overall, the Cronbach’s alpha for *overall digital health literacy for vaccine score* is 0.92 while the four subscales’ reliability range from 0.73 to 0.88.

| DHL Vaccine Scale | Mean | SD | n | $alpha$ | Number of items |
| --- | --- | --- | --- | --- | --- |
| *Overall digital health literacy for vaccine score (4 subscales)* | **2.93** | 0.58 | 4589 | 0.92 | 12 |
| *Subscale 1: Information seeking* | **2.98** | 0.69 | 4691 | 0.87 | 3 |
| dl21. Choose from all the information you find? | 2.97 | 0.77 | 4692 |  |  |
| dl22. Use the appropriate words or search query to find the information you are looking for? | 3.03 | 0.74 | 4694 |  |  |
| dl23. Find the exact information you’re looking for? | 2.92 | 0.79 | 4693 |  |  |
| *Subscale 2: Adding self-generated content* | **2.84** | 0.65 | 4611 | 0.73 | 3 |
| d51. Clearly formulate yours health-related question or concern? | 2.81 | 0.78 | 4632 |  |  |
| d52. Express your opinion, thoughts or feelings in writing? | 2.85 | 0.81 | 4627 |  |  |
| d53. Write your message that way, so that people understand exactly what you mean | 2.85 | 0.81 | 4622 |  |  |
| *Subscale 3: Evaluating reliability* | **2.88** | 0.74 | 4682 | 0.86 | 3 |
| dl24. Decide if the information is reliable or not? | 2.85 | 0.86 | 4693 |  |  |
| dl25. Decide if the information is written with commercial interests | 2.84 | 0.84 | 4686 |  |  |
| dl26. Check different websites to see if they provide the same information? | 2.95 | 0.80 | 4689 |  |  |
| *Subscale 4: Determining relevance* | **3.00** | 0.70 | 4684 | 0.88 | 3 |
| dl27. Decide if the information you’ve found applies to you? | 2.95 | 0.79 | 4689 |  |  |
| dl28. Apply the information you found in your daily life? | 2.99 | 0.78 | 4686 |  |  |
| dl29. Use the information you’ve found to make decisions about your health | 3.06 | 0.78 | 4688 |  |  |

## Appendix 2

Digital Health Literacy subscales asked in the survey were as follows:

1. Subscale 1: Information Seeking (Q2) *When you search the Internet for information about the COVID-19 vaccine or related topics, how easy or difficult is it for you to…*
   - dl21: …choose from all the information you find?
   - dl22: …use the appropriate words or search query to find the information you are looking for?
   - dl23: …find the exact information you’re looking for?

- Table: Subscale 1: Information seeking

|  | n | % |
| --- | --- | --- |
|  | | |
| [... to choose from all the information you find?] | | |
| Very difficult | 130 | 2.8 |
| Difficult | 1,064 | 22.7 |
| Easy | 2,313 | 49.3 |
| Very easy | 1,185 | 25.3 |
| Total | 4,692 | 100.0 |
|  | | |
| [... to use the appropriate words or search query to find the information you ar | | |
| Very difficult | 126 | 2.7 |
| Difficult | 834 | 17.8 |
| Easy | 2,491 | 53.1 |
| Very easy | 1,243 | 26.5 |
| Total | 4,694 | 100.0 |
|  | | |
| [… Finding the exact information you're looking for?] | | |
| Very difficult | 173 | 3.7 |
| Difficult | 1,157 | 24.7 |
| Easy | 2,222 | 47.3 |
| Very easy | 1,141 | 24.3 |
| Total | 4,693 | 100.0 |

1. Subscale 2: Adding self-generated content (Q5) *When typing a message (for example, in a forum or on social media like Facebook or Twitter) about the COVID-19 vaccine or related topics, it’s easy or difficult for you to…*
   - d51: …clearly formulate yours health-related question or concern?
   - d52: …express your opinion , thoughts or feelings in writing?
   - d53: …write your message that way, so that people understand exactly what you mean

- Table: Subscale 2: Adding self-generated content

|  | n | % |
| --- | --- | --- |
|  | | |
| [... to clearly formulate yours health-related question or concern?] | | |
| Very difficult | 227 | 4.9 |
| Difficult | 1,278 | 27.6 |
| Easy | 2,292 | 49.5 |
| Very easy | 835 | 18.0 |
| Total | 4,632 | 100.0 |
|  | | |
| [... express your opinion , thoughts or feelings in writing?] | | |
| Very difficult | 269 | 5.8 |
| Difficult | 1,109 | 24.0 |
| Easy | 2,304 | 49.8 |
| Very easy | 945 | 20.4 |
| Total | 4,627 | 100.0 |
|  | | |
| [... to write your message that way, so that people understand exactly what you. | | |
| Very difficult | 244 | 5.3 |
| Difficult | 1,164 | 25.2 |
| Easy | 2,238 | 48.4 |
| Very easy | 976 | 21.1 |
| Total | 4,622 | 100.0 |

1. Subscale 3: Evaluating reliability (Q2) *When you search the Internet for information about the COVID-19 vaccine or related topics, how easy or difficult is it for you to…*
   - dl24: …decide if the information is reliable or not?
   - dl25: …decide if the information is written with commercial interests (eg, by people trying to sell a product)?
   - dl26: …check different websites to see if they provide the same information?

- Table: Subscale 3: Evaluating reliability

|  | n | % |
| --- | --- | --- |
|  | | |
| [... to decide if the information is reliable or not?] | | |
| Very difficult | 258 | 5.5 |
| Difficult | 1,357 | 28.9 |
| Easy | 1,921 | 40.9 |
| Very easy | 1,157 | 24.7 |
| Total | 4,693 | 100.0 |
|  | | |
| [... to decide if the information is written with commercial interests] | | |
| Very difficult | 235 | 5.0 |
| Difficult | 1,362 | 29.1 |
| Easy | 1,990 | 42.5 |
| Very easy | 1,099 | 23.5 |
| Total | 4,686 | 100.0 |
|  | | |
| […Check different websites to see if they provide the same information?] | | |
| Very difficult | 180 | 3.8 |
| Difficult | 1,075 | 22.9 |
| Easy | 2,220 | 47.3 |
| Very easy | 1,214 | 25.9 |
| Total | 4,689 | 100.0 |

1. Subscale 4: Determining relevance (Q2) *When you search the Internet for information about the COVID-19 vaccine or related topics, how easy or difficult is it for you to…*
   - dl27: …decide if the information you’ve found applies to you?
   - dl28: …apply the information you found in your daily life?
   - dl29: …use the information you’ve found to make decisions about your health (eg, measures of protection, hygiene regulations)

- Table: Subscale 4: Determining relevance

|  | n | % |
| --- | --- | --- |
|  | | |
| [... to decide if the information you've found applies to you?] | | |
| Very difficult | 167 | 3.6 |
| Difficult | 1,068 | 22.8 |
| Easy | 2,267 | 48.3 |
| Very easy | 1,187 | 25.3 |
| Total | 4,689 | 100.0 |
|  | | |
| [... to apply the information you found in your daily life?] | | |
| Very difficult | 165 | 3.5 |
| Difficult | 933 | 19.9 |
| Easy | 2,358 | 50.3 |
| Very easy | 1,230 | 26.2 |
| Total | 4,686 | 100.0 |
|  | | |
| [… Use the information you've found to make decisions about your health?] | | |
| Very difficult | 138 | 2.9 |
| Difficult | 891 | 19.0 |
| Easy | 2,231 | 47.6 |
| Very easy | 1,428 | 30.5 |
| Total | 4,688 | 100.0 |

Appendix 3: Adjusted ORs for sufficient total DHL score (model 1) and sufficient DHL subscales scores (model 2)

|  | (Model 1) | (Model 2) |
| --- | --- | --- |
|  | DHL_sufficient | DHL_subscales_sufficient |
| Outcome: Intention for vaccination |  |  |
| *DHL total* |  |  |
| Insufficient DHL | 1.00 |  |
| Sufficient DHL | 1.64^***^[1.41,1.90] |  |
| *Sufficient_S1 vs insufficient* |  | 1.12 [0.92,1.36] |
| *Sufficient_S2 vs insufficient* |  | 1.10 [0.92,1.30] |
| *Sufficient_S3 vs insufficient* |  | 1.16 [0.95,1.42] |
| *Sufficient_S4 vs insufficient* |  | 1.48^***^ [1.21,1.80] |
| *Age group* |  |  |
| 18-29 | 1.00 | 1.00 |
| 30-49 | 0.87[0.69,1.08] | 0.87[0.70,1.09] |
| 50 and above | 1.18[0.82,1.72] | 1.23[0.85,1.79] |
| *Sex* |  |  |
| Female | 1.00 | 1.00 |
| Male | 1.22^**^[1.04,1.42] | 1.21^**^[1.04,1.41] |
| *Education* |  |  |
| Up to secondary level | 1.00 | 1.00 |
| Tertiary education level | 1.47^***^[1.25,1.73] | 1.46^***^[1.24,1.71] |
| *Country* |  |  |
| Bangladesh | 1.00 | 1.00 |
| Brazil | 14.42^***^[5.90,35.23] | 12.94^***^[5.27,31.77] |
| Egypt | 1.32[0.76,2.29] | 1.27[0.73,2.21] |
| Indonesia | 5.71^***^[3.45,9.43] | 5.30^***^[3.19,8.81] |
| Iran | 2.92^***^[1.79,4.77] | 2.89^***^[1.76,4.75] |
| Malaysia | 1.68^***^[1.15,2.47] | 1.64^**^[1.12,2.41] |
| Myanmar | 1.61[0.84,3.12] | 1.52[0.79,2.93] |
| Philippines | 0.69^*^[0.47,1.00] | 0.67^**^[0.45,0.99] |
| Thailand | 6.09^***^[2.88,12.87] | 5.47^***^[2.57,11.67] |
| Turkey | 33.53^***^[17.07,65.87] | 31.00^***^[15.66,61.35] |
| United Arab Emirates | 1.31[0.85,2.01] | 1.23[0.80,1.90] |
| Other | 0.77[0.47,1.25] | 0.74[0.45,1.20] |
| *Area of residence* |  |  |
| Rural | 1.00 | 1.00 |
| Urban | 1.15^*^[0.97,1.37] | 1.16^*^[0.98,1.38] |
| *Employment status* |  |  |
| Not working | 1.00 | 1.00 |
| Working | 1.12[0.92,1.38] | 1.13[0.92,1.39] |
| Student | 1.92^***^[1.43,2.59] | 1.91^***^[1.42,2.57] |
| Other | 0.76^**^[0.59,0.97] | 0.76^**^[0.59,0.97] |
| *Income* |  |  |
| Insufficient income | 1.00 | 1.00 |
| Sufficient income | 1.50^***^[1.27,1.76] | 1.47^***^[1.25,1.74] |
|  |  |  |
| Observations | 4553 | 4553 |
| Pseudo *R*^2^ | 0.181 | 0.185 |
| Hosmer-Lemeshow chi-square | .397 | .953 |

Explanatory variables in model 1: DHL total, age, sex, education, country, urban/rural, employment status and income

Explanatory variables in model 2: DHL subscales1, subscales2, subscales 3, subscales4, age, sex, education, country, urban/rural, employment status and income

^#^The outcome variable is “intention for vaccination”, Yes=1, No/Don’t know=0 (reference)

^*^ p<0.05, ^**^ p <0.01, ^***^ p <0.001
